# Systemic Immune-Inflammation Index Predicts the Clinical Outcome in Patients with mCRPC Treated with Abiraterone

**DOI:** 10.3389/fphar.2016.00376

**Published:** 2016-10-13

**Authors:** Cristian Lolli, Orazio Caffo, Emanuela Scarpi, Michele Aieta, Vincenza Conteduca, Francesca Maines, Emanuela Bianchi, Francesco Massari, Antonello Veccia, Vincenzo E. Chiuri, Gaetano Facchini, Ugo De Giorgi

**Affiliations:** ^1^Medical Oncology Department, Istituto Scientifico Romagnolo per lo Studio e la Cura dei Tumori, Istituto di Ricovero e Cura a Carattere ScientificoMeldola, Italy; ^2^Medical Oncology Department, Santa Chiara HospitalTrento, Italy; ^3^Medical Oncology Department, Istituto di Ricovero e Cura a Carattere Scientifico Centro di Riferimento Oncologico della BasilicataRionero in Vulture, Italy; ^4^Medical Oncology Unit, Infermi HospitalRimini, Italy; ^5^Division of Oncology, S.Orsola-Malpighi HospitalBologna, Italy; ^6^Medical Oncology Unit, Vito Fazzi HospitalLecce, Italy; ^7^Division of Medical Oncology, Department of Uro-Gynaecological Oncology, Istituto Nazionale Tumori “Fondazione G. Pascale” – Istituto di Ricovero e Cura a Carattere ScientificoNaples, Italy

**Keywords:** systemic immune-inflammation index, PSA, CRPC, prostate cancer, prognostic factor, abiraterone

## Abstract

**Background:** A systemic immune-inflammation index (SII) based on neutrophil (*N*), lymphocyte (*L*), and platelet (*P*) counts has shown a prognostic impact in several solid tumors. The aim of this study is to evaluate the prognostic role of SII in metastatic castration-resistant prostate cancer (mCRPC) patients treated with abiraterone post docetaxel.

**Patients and Methods:** We retrospectively reviewed consecutive mCRPC patients treated with abiraterone after docetaxel in our Institutions. X-tile 3.6.1 software, cut-off values of SII, neutrophil-to-lymphocyte ratio (NLR) defined as N/L and platelets-to-lymphocyte ratio (PLR) as P/L. Overall survival (OS) and their 95% Confidence Intervals (95% CI) was estimated by the Kaplan-Meier method and compared with the log-rank test. The impact of SII, PLR, and NLR on overall survival (OS) was evaluated by Cox regression analyses and on prostate-specific antigen (PSA) response rates were evaluated by binary logistic regression.

**Results:** A total of 230 mCRPC patients treated abiraterone were included. SII ≥ 535, NLR ≥ 3 and PLR ≥ 210 were considered as elevated levels (high risk groups. The median OS was 17.3 months, 21.8 months in SII < 535 group and 14.7 months in SII ≥ 535 (*p* < 0.0001). At univariate analysis Eastern Cooperative Oncology Group (ECOG) performance status, previous enzalutamide, visceral metastases, SII, NLR, and PLR predicted OS. In multivariate analysis, ECOG performance status, previous enzalutamide, visceral metastases, SII, and NLR remained significant predictors of OS [hazard ratio (HR) = 5.08, *p* < 0.0001; HR = 2.12, *p* = 0.009, HR = 1.77, 95% *p* = 0.012; HR = 1.80, *p* = 0.002; and HR = 1.90, *p* = 0.001, respectively], whereas, PLR showed a borderline ability only (HR = 1.41, *p* = 0.068).

**Conclusion:** SII and NLR might represent an early and easy prognostic marker in mCRPC patients treated with abiraterone. Further studies are needed to better define their impact and role in these patients.

## Introduction

Prostate cancer is the most common cancer and the second cause of death for cancer in men (Siegel et al., 2016). Castration resistance is an extremely heterogeneous condition ranging from asymptomatic patients with only an initial increase of prostate-specific antigen (PSA) to patients with high-volume of disease and potentially heavily symptomatic (Attard et al., 2016). In the last years, new agents prolonged survival and improved quality of life of patients.with metastatic castration-resistant prostate cancer (mCRPC), including abiraterone, enzalutamide, cabazitaxel, sipuleucel T, and radium-223 (de Bono et al., 2010, 2011; Kantoff et al., 2010; Scher et al., 2012; Parker et al., 2013). In order to better select treatment and sequencing of these drugs, the identification of prognostic and predictive factors is increasingly investigated.

The effect of inflammation in prognosis and progression has been shown in patients with mCRPC, and peripheral blood circulating cells (neutrophils, lymphocytes and platelets) included in inflammation indices have been associated with prognosis in these patients (De Marzo et al., 2007; Brennen et al., 2013; Leibowitz-Amit et al., 2014; Lorente et al., 2015; Conteduca et al., 2016). Neutrophil-to-lymphocyte ratio (NLR) and platelets-to-lymphocyte ratio (PLR) are markers of inflammation associated with prognosis in several cancers (Templeton et al., 2014; Cannon et al., 2015; Rossi et al., 2015). Recently, NLR has shown a prognostic role in patients with mCRPC also when treated with abiraterone, enzalutamide, docetaxel, or cabazitaxel (Leibowitz-Amit et al., 2014; Nuhn et al., 2014; Lorente et al., 2015; Conteduca et al., 2016). Systemic immune-inflammation index (SII), a novel inflammatory index based on neutrophil, lymphocyte, and platelet counts, has recently emerged as a powerful prognostic index in hepatocarcinoma, renal cell cancer, and colorectal (Hu et al., 2014; Lolli et al., 2016; Passardi et al., 2016), but to date no data have been reported in mCRPC.

In this retrospective analysis, we aimed to evaluate prognostic implications of SII at baseline and changes during treatment with abiraterone in patients with mCRPC post-docetaxel. In addition, we considered also NLR and PLR value and their respective changes at 4 weeks to try to better define the impact of these inflammatory indexes on mCRPC patients treated with abiraterone.

## Patients and Methods

We retrospectively evaluated 230 patients with mCRPC treated with abiraterone in the post docetaxel setting between April 2011 and May 2015 in our seven Institutions. SII, the inflammatory index based on platelets (*P*), neutrophils (*N*), and lymphocytes (*L*) counts, was defined as *P* ×*N/L*. In order to determine the cutoff value of SII, NLR defined as *N*/*L* and PLR as *P*/*L* at the baseline data, the X-tile 3.6.1 software (Yale University, New Haven, CT, USA) was used.

Data were collected into electronic data files by the local physicians and checked at the central data management. Patients not treated with docetaxel before abiraterone (pre chemotherapy setting) were not considered for the analysis.

Abiraterone was administered according to clinical practice at the dose of 1000 mg once daily and was associated with prednisone 5 mg BID. Patients were treated until disease progression or unacceptable toxicity occurred. All patients provided written informed consent. All recorded PSA test, full blood examinations, including a complete blood count, and scan results were retrospectively collected for these patients, evaluated commonly every 4 weeks for serologic PSA response and as clinically indicated for imaging assessment. PSA response was defined according to the Prostate Cancer Working Group 2 criteria as a decline on serum PSA levels during abiraterone treatment of 50% or more from PSA baseline value maintained for ≥3 weeks (Scher et al., 2008). In addition, the PSA decline of 30% or more after 4 weeks of treatment only has been recently demonstrated as a useful predictive marker and was considered for comparison with early inflammatory changes (Rescigno et al., 2016). Monthly PSA measurements were carried out during the first 3 months of abiraterone, and thereafter every 1–3 months according to physicians’ discretion. A clinical deterioration or a radiologic evidence of PD as well as PSA increase associated with therapy interruption secondary to unacceptable toxicity or death were sufficient to establish abiraterone discontinuation. This study was carried out in accordance with the approval of the local ethical committee.

### Primary Objective and Statistical Analysis

The primary objective of this study was to evaluate the ability of SII to predict the overall survival (OS) in patients with mCRPC treated with abiraterone in post-docetaxel setting. As patients were treated in clinical practice, radiological assessment was not carried out at pre-determined intervals in most patients, so that radiological progression-free survival (PFS) could not be assessed for all patients.

Data were summarized by frequency for categorical variables and by median and range for continuous variables. Association between categorical variables was assessed using the χ^2^ or Fisher’s exact test. Differences were considered statistically significant when *p* < 0.05. OS was calculated from the start of abiraterone until death or last follow-up. The Kaplan–Meier method was used to estimate OS. The log-rank test and Cox proportional hazard regression were used to test for differences between groups. After univariate analysis, a multivariate analysis was carried out by Cox regression model. Estimated hazard ratios (HRs), their 95% confidence intervals (95% CI), and *p* values were calculated from the Cox proportional hazard regression models. The impact of inflammatory index conversion on survival outcomes was evaluated by the landmark analysis at 4 weeks. All statistical analyses were carried out with SAS statistical software, version 9.4 (SAS Institute, Cary, NC, USA).

## Results

A total of 230 mCRPC patients with a median age of 74 years (range, 45–90 years) underwent abiraterone treatment post-docetaxel were included in the study. Among all patients, 35 (15.2%) had visceral metastases, 21 (9.1%) received previous enzalutamide. Patient characteristics at baseline are reported in **Table 1**. The optimal cutoff point was calculated by X-tile 3.6.1 software to determine the best threshold of indices levels to predict OS at 18 months, and for the SII was 535 × 10^9^, for NLR was 3 and PLR was 210. Then, SII ≥ 535, NLR ≥ 3, and PLR ≥ 210 were considered as elevated levels (high-risk groups). Visceral metastases were not associated with SII groups (*p* = 0.479), whereas a trend versus an association with NLR and PLR groups was reported (*p* = 0.057 and *p* = 0.055, respectively).

**Table 1:** Patient characteristics.

	Total (*N* = 230) No. (%)
**Age**	
Median value (range), years	74 (45-90)
**ECOG performance status**	
0	102 (44.3)
1	115 (50.0)
2	13 (5.7)
**Gleason score**	
<8	75 (36.8)
≥8	129 (63.2)
Unknown/missing	26
**Sites of metastasis**	
Bone only	85 (37.0)
Nodal only	22 (9.6)
Bone+nodal	88 (38.3)
Visceral	35 (15.2)
**Previous chemotherapy**	
1 line	170 (73.9)
>1 line	60 (26.1)
**Previous enzalutamide**	
No	209 (90.9)
Yes	21 (9.1)

PSA decline ≥50% during treatment from the baseline value (PSA response) was assessable in 229 (99.6%) of 230 patients, while PSA decline ≥30% at 4 weeks of treatment (early PSA decline ≥30%) was evaluable in 211 (91.7%) cases. PSA response was reported in 92 (40.2%) of 229 evaluable patients, while early PSA decline ≥30% was reported in 93 (44.1%) of 211 evaluable patients. No association was observed among PSA response and SII and NLR, with a trend for PLR (*p* = 0.126, *p* = 0.709, and *p* = 0.057, respectively), whereas an association with early PSA decline ≥30% was observed for PLR (*p* = 0.0004), a trend was seen for SII (*p* = 0.081), with no association with NLR (*p* = 0.357). We also divided up the two baseline groups (baseline high risk and low risk) of the three parameters (SII, NLR, and PLR) on the basis of the values at 4 week (4-week high risk and low risk), obtaining four subgroups: low–low, low–high, high–low, and high–high. We then compared the 4-week conversion of SII, NLR, and PLR groups with PSA response and early PSA decline ≥30%. PSA response was not associated with 4-week conversions of NLR (*p* = 0.603), but a trend was observed for SII, and PLR (*p* = 0.055 and *p* = 0.073, respectively), whereas early PSA decline ≥30% was associated with 4-week conversion of SII and PLR (*p* = 0.016 and *p* = 0.0003, respectively) and a trend was observed with 4-week conversion of NLR (*p* = 0.114).

**FIGURE 1:**
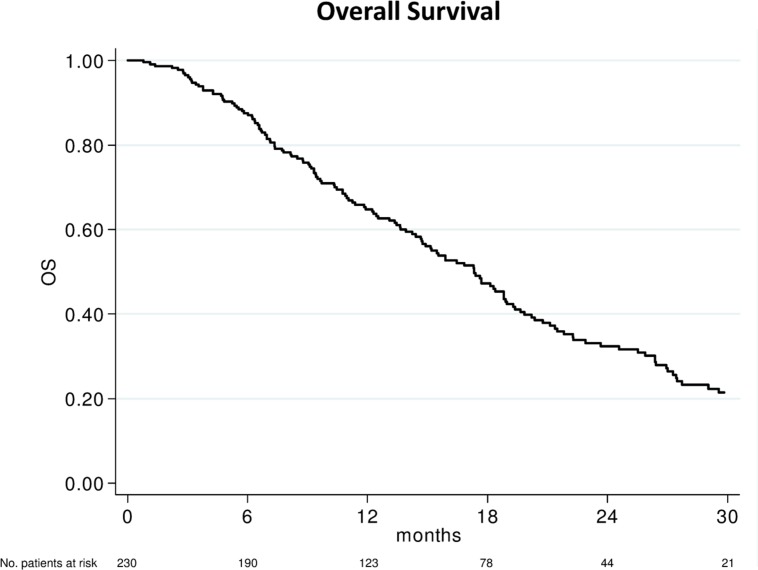
**Kaplan–Meier plots illustrating overall survival in the patient population**.

**FIGURE 2:**
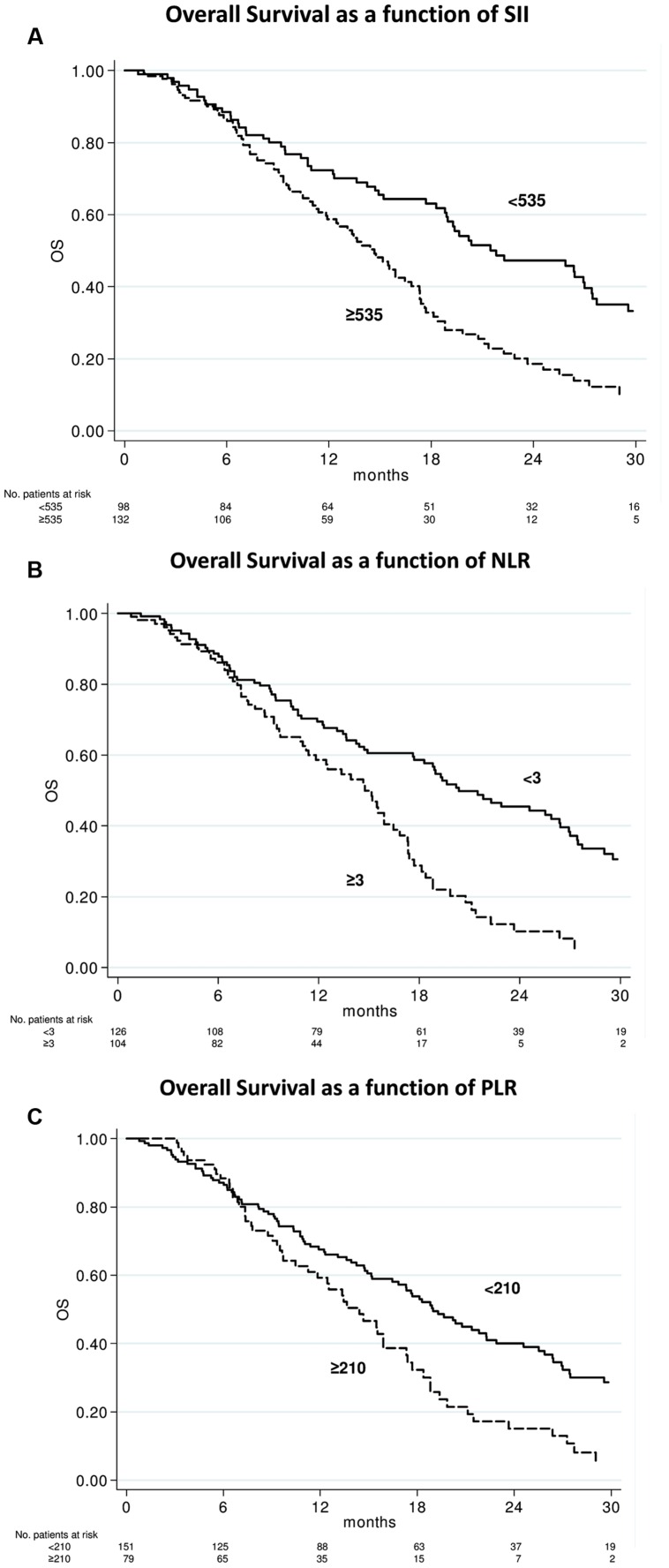
**Overall survival according to baseline systemic immune-inflammation index (SII), neutrophil-to-lymphocyte ratio (NLR), and platelets-to-lymphocyte ratio (PLR) indexes.** Kaplan–Meier plots illustrating overall survival according to baseline SII ≥ 535 vs. < 535 (*p* < 0.0001) **(A)**, NLR ≥ 3 vs. < 3 (*p* < 0.0001) **(B)**, and PLR ≥ 210 vs. < 210 (*p* = 0.002) **(C)**.

After a median follow-up of 29 months (range, 1–55), 156 (67.8%) had died. The median OS was 17.3 months (95% CI 14.7–18.9; **Figure 1**). In relation to SII value, the median OS was 21.8 months (95 % CI 18.8–27.4) and 14.7 months (95% CI 11.9–16.8) (*p* < 0.0001) in patients with baseline SII < 535 or ≥535, respectively (**Figure 2A**). In relation to NLR value, the median OS was 20.4 months (95% CI 17.7–26.4) and 14.7 months (95% CI 11.4–16.4; *p* < 0.0001) in patients with baseline NLR < 3 or ≥3, respectively (**Figure 2B**). In relation to PLR value, the median OS was 19.0 months (95% CI 16.4–22.3) and 14.4 months (95% CI 11.2–17.3; *p* = 0.002) in patients with baseline PLR < 210 or ≥210, respectively (**Figure 2C**). A univariate analysis revealed that Eastern Cooperative Oncology Group (ECOG) performance status, previous enzalutamide, presence of visceral metastases and all inflammatory indexes (SII, NLR, and PLR) were significant predictors of OS (**Table 2**). In multivariate analysis, among clinical variables, ECOG performance status, previous enzalutamide therapy and presence of visceral metastases remained significant predictors of OS (HR = 5.08, 95% CI 2.34–10.99, *p* < 0.0001; HR = 2.12, 95% CI 1.20–3.73, *p* = 0.009, and HR = 1.77, 95% CI 1.13–2.75, *p* = 0.012, respectively); whereas, among inflammatory indexes, SII and NLR remained as predictor of OS (HR = 1.80, 95% CI 1.23–2.62, *p* = 0.002; and HR = 1.90, 95% CI 1.30–2.79, *p* = 0.001, respectively) whereas PLR showed a borderline ability only (HR = 1.41, 95% CI 0.97–2.03, *p* = 0.068).

**Table 2:** Univariate analysis.

	Overall Survival				
Variables	No. patients	No. events	Median Overall survival (OS) (months) (95% CI)	Hazard ratio (HR) (95% CI)	*p*-value
**Overall**	230	156	17.3 (14.7-18.9)	–	–
**Age**	–	–	–	0.998 (0.977–1.020)	0.860
**Gleason score**					
<8	75	52	15.2 (12.2–20.4)	1.00	
≥8	129	85	17.4 (13.7–19.4)	0.91 (0.65–1.29)	0.612
**ECOG performance status**					
0–1	217	145	17.7 (15.2–19.6)	1.00	
≥2	13	11	8.7 (1.1–11.4)	3.52 (1.89–6.55)	<0.0001
**Sites of metastases**					
No visceral	195	130	18.1 (15.9–19.8)	1.00	
Visceral	35	26	10.5 (7.0–15.5)	1.64 (1.07–2.50)	0.022
**Previous chemotherapy treatment**					
1 line	170	109	16.8 (13.9–18.8)	1.00	
>1 line	60	47	18.3 (13.7–21.8)	0.79 (0.55–1.12)	0.188
**Previous enzalutamide**					
No	209	140	17.7 (15.2–19.6)	1.00	
Yes	21	16	7.0 (5.2–15.5)	1.99 (1.18–3.35)	0.010
**Neutrophil-to-lymphocyte ratio (NLR)**					
<3	126	85	20.4 (17.7–26.4)	1.00	
≥3	104	71	14.7 (11.4–16.4)	2.13 (1.51–2.99)	<0.0001
**Platelets-to-lymphocyte ratio (PLR)**					
<210	151	100	19.0 (16.4–22.3)	1.00	
≥210	79	56	14.4 (11.2–17.3)	1.68 (1.20–2.35)	0.002
**SII**					
<535	98	64	21.8 (18.8–27.4)	1.00	
≥535	132	92	14.7 (11.9–16.8)	2.08 (1.48–2.92)	<0.0001

We studied the above-mentioned four conversion subgroups of SII, NLR and PLR for OS. The high–high group showed a significantly worse OS than low–low group with all inflammatory indices, whereas results in the low–high and high–low groups remained less significant (**Table 3**).

**Table 3:** Changes in indices and clinical outcome.

	Overall Survival					
Variables	n. patients	n. events	Median OS (months) (95% CI)	HR (95% CI)	*p*-value
**NLR**					
Low–Low (<3–<3)	107	70	21.8 (17.6-27.0)	1.00	
Low–High(<3–≥3)	19	15	18.8 (12.2-29.0)	1.49 (0.84–2.63)	0.169
High–Low(≥3–<3)	22	20	13.4 (7.8-15.9)	3.22 (1.89–5.49)	<0.0001
High–High (≥3–≥3)	81	50	15.5 (11.8-18.4)	2.04 (1.39–3.01)	0.0003
**PLR**					
Low–Low (<210–<210)	142	92	19.3 (15.2-24.6)	1.00	
Low–High (<210–≥210)	8	7	18.6 (2.2-30.1)	1.54 (0.71–3.33)	0.274
High–Low (≥210–<210)	28	21	13.7 (9.5-18.8)	1.46 (0.91–2.36)	0.116
High–High (≥210–≥210)	51	35	14.4 (9.7-17.4)	2.01 (1.34–3.01)	0.0008
**SII**					
Low–Low (<535–<535)	79	50	25.9 (18.8-29.6)	1.00	
Low–High (<535–≥535)	19	14	19.4 (14.2-27.0)	1.31 (0.72–2.38)	0.372
High–Low (≥535–<535)	24	15	16.4 (10.9-18.1)	1.79 (0.99–3.22)	0.054
High–High (≥535–≥535)	107	76	13.9 (11.4-17.3)	2.29 (1.57–3.33)	<0.0001

## Discussion

Our results confirmed the activity of abiraterone in post-docetaxel setting in routinely clinical practice with a PSA response rate of 44.5% and a median OS of 17.3 months comparable to results of the pivotal phase III clinical trial (de Bono et al., 2011). The prognostic significance of NLR, PLR, and percentage of lymphocytes evaluate before systemic treatment was shown in several tumors (De Giorgi et al., 2012; Templeton et al., 2014; Cannon et al., 2015; Rossi et al., 2015), while SII to date has been evaluated before antiangiogenic agents only in CRC and RCC (Lolli et al., 2016; Passardi et al., 2016). Herein, we reported for the first time SII to be an independent predictor of OS for patients with mCRPC treated with abiraterone, a recently approved hormonal therapy in mCRPC. Its ability to predict OS was higher than conventional parameters such as Gleason score (**Table 2**). We also confirmed previous data on the OS prediction ability of NLR in these patients (Leibowitz-Amit et al., 2014), whereas PLR showed a borderline significance in the multivariate analysis (HR = 1.41, *p* = 0.068). Our results suggest a possible prominent prognostic role for neutrophil levels, included in SII and NLR, but not in PLR. In our study, there was not a significant predictive role of these inflammatory indexes for PSA response. In a previous study, other authors analyzed the impact of NLR in mCRPC patients treated with abiraterone showing ability to predict PSA response and OS. However, the authors established the PSA response as the primary objective instead of OS, which was not used since most patients were alive at the time of that analysis (Leibowitz-Amit et al., 2014). The continuous variable NLR was dichotomized using optimal cutoffs of NLR = 5, which retained association with the primary end point, the PSA response, by univariate analysis. In our study, 156 (67.8%) of 230 patients had died at the time of analysis, then we used OS as primary end point, and performed ROC curve analysis to determine the best threshold of indices levels to predict OS at 18 months; ROC curves identified the cut-off values for NLR of 3. This difference in the definition of the NLR threshold could have at least in part influenced the different ability of NLR to predict PSA response in each study. In addition, other limitations should be recognized as the retrospective nature of the analysis and the relative under-representation of some groups as ECOG performance status = 2 (15%) and patients pre-treated with enzalutamide (9%). Another limiting factor is missing validation group of patients, thus the study should be considered as hypothesis generating for next larger and/or prospective studies.

We also evaluate the potential role of 4-week changes of SII, NLR, and PLR on the prognosis (**Table 3**). The high–high groups showed a significantly worse OS than low–low groups with all inflammatory indices, whereas results in the low–high and high–low groups remained more controversial, even if these latest groups were poorly represented counting from 3.5 to 12.2% in all three inflammatory indexes (**Table 3**). Another possible limitation for the interpretation of these early changes may be the use of prednisone 5 mg BID in combination with abiraterone, which could influence these inflammatory indexes. In a large study in 755 patients with mCRPC treated with chemotherapy and prednisone 5 mg BID, the conversion of NLR counts from high–low had a significant impact on the OS, whereas the change from low to high did not modify the prognosis (Lorente et al., 2015). There is lacking information in the literature on the association of inflammatory indexes and poor prognosis variants of mCRPC, as presence of visceral metastases, neuroendocrine differentiation, AR gene amplification or mutations (Burgio et al., 2014; Conteduca et al., 2014, 2015; Azad et al., 2015; Salvi et al., 2015). In our study the presence of visceral metastases, even if poorly represented (*n* = 35, 15.2%), was initially associated with NLR and PLR poor prognosis groups (*p* = 0.057 and *p* = 0.055, respectively), showing possible correlations between systemic inflammatory activation and more aggressive mCRPC behavior.

In the last decade, circulating tumor cell (CTC) counts has emerged as a biomarker for assessing prognosis and treatment outcome in mCRPC (de Bono et al., 2008), Moreover, post-treatment CTC changes has been associated with mCRPC survival, and it has been shown that CTC count has superior performance to other circulating biomarkers including PSA (Lorente et al., 2016).

The prognostic role of the SII might be elucidated by the function of peripheral N, L, and P and the their relationship with CTC (De Giorgi et al., 2016; Mego et al., 2016). Our results suggest the SII and NLR could be a more objective marker than indexes such as the PLR. A better understanding of the role of N, L, and P will help elucidate the association between cancer, inflammation, and immunity.

The SII biomarker could represent a novel predictive marker of clinical outcome to other treatments such as chemotherapy, radium, radiotherapy, and should be tested in these clinical situations.

A potential role for new immune check-point inhibitors in combination with new hormonal therapies in mCRPC has been recently suggested (Bishop et al., 2015; Graff et al., 2016), then the identification of useful inflammatory-immune biomarkers will be crucial for the development of these new drugs for these patients (Saylor et al., 2012). The impact of immune-inflammation indexes at baseline and during abiraterone (and possibly enzalutamide) underlie a potential increase of immune-inflammation biomarkers during these treatments. Potential use of these biomarkers to predict the effect of immune-check-point inhibitors alone or in combination with abiraterone in CRPC.

## Conclusion

Inflammation deserves a pivotal role in prostate cancer carcinogenesis and progression. Our results suggest that SII and NLR can provide clinically meaningful information and might represent an early and easy prognostic marker needing further studies in mCRPC patients.

## Author Contributions

CL and UG designed the study and wrote the manuscript. CL and VC collected data. ES performed the statistical analysis. OC, MA, FMai, EB, FMas, AV, VC, and GF contributed to clinical data collection. All authors revised and approved the manuscript.

## Conflict of Interest Statement

The authors declare that the research was conducted in the absence of any commercial or financial relationships that could be construed as a potential conflict of interest.
